# A Novel Form of Memory for Auditory Fear Conditioning at a Low-Intensity Unconditioned Stimulus

**DOI:** 10.1371/journal.pone.0004157

**Published:** 2009-01-09

**Authors:** Ayumi Kishioka, Fumiaki Fukushima, Tamae Ito, Hirotaka Kataoka, Hisashi Mori, Toshio Ikeda, Shigeyoshi Itohara, Kenji Sakimura, Masayoshi Mishina

**Affiliations:** 1 Department of Molecular Neurobiology and Pharmacology, Graduate School of Medicine, University of Tokyo, Tokyo, Japan; 2 Laboratory of Behavioral Genetics, Brain Science Institute, RIKEN, Saitama, Japan; 3 Department of Cellular Neurobiology, Brain Research Institute, Niigata University, Niigata, Japan; Rutgers University, United States of America

## Abstract

Fear is one of the most potent emotional experiences and is an adaptive component of response to potentially threatening stimuli. On the other hand, too much or inappropriate fear accounts for many common psychiatric problems. Cumulative evidence suggests that the amygdala plays a central role in the acquisition, storage and expression of fear memory. Here, we developed an inducible striatal neuron ablation system in transgenic mice. The ablation of striatal neurons in the adult brain hardly affected the auditory fear learning under the standard condition in agreement with previous studies. When conditioned with a low-intensity unconditioned stimulus, however, the formation of long-term fear memory but not short-tem memory was impaired in striatal neuron-ablated mice. Consistently, the ablation of striatal neurons 24 h after conditioning with the low-intensity unconditioned stimulus, when the long-term fear memory was formed, diminished the retention of the long-term memory. Our results reveal a novel form of the auditory fear memory depending on striatal neurons at the low-intensity unconditioned stimulus.

## Introduction

Fear is one of the most potent emotional experiences of our lifetime and is an adaptive component of response to potentially threatening stimuli, serving a function that is critical to the survival of higher vertebrates [1], [2]. Too much or inappropriate fear, however, accounts for many common psychiatric problems [3]–[5]. A fearful experience can establish an emotional memory that results in permanent behavioral changes and emotional memories have been observed in many animal groups [6]. The brain mechanisms underlying fear are similar in different species and the fear system will respond similarly in a person or a rodent, using a limited set of defense response strategies [7]. The memory of learned fear can be assessed quantitatively using a Pavlovian fear-conditioning paradigm [1], [2]. During fear conditioning, an initially neutral conditioned stimulus (CS, e.g. an auditory tone) acquires biological significance by becoming associated with an aversive unconditioned stimulus (US, e.g. a footshock). After learning this association, an animal responds to the previously neutral CS with a set of defensive behavioral responses, such as freezing. Anatomical tracing and lesion studies demonstrated the importance of the amygdala for fear conditioning [8]–[10]. Subsequent physiological experiments showed that learning produces prolonged synaptic modification in both of the inputs to the amygdala: the thalamo-amygdala pathway [11], [12] and the cortico-amygdala pathway [13]. Evidence from many studies suggests that the amygdala—in particular, the lateral/basolateral nuclei—plays an essential role in the acquisition, storage and expression of fear memory [1], [7], [14]–[18].

Here, we developed an inducible striatal neuron ablation system in transgenic mice and examined the effect of striatal neuron ablation on auditory fear conditioning with different intensities of US. Under the standard condition, the ablation of striatal neurons in the adult brain hardly affected the auditory fear conditioning in agreement with previous studies [18]–[22]. We found, however, that under a weak condition, the formation of long-term auditory fear memory but not short-term memory was impaired by the ablation of striatal neurons. Our results suggest the presence of two forms of auditory fear memories distinguished by the US intensity and by the requirement of striatal neurons. Our finding that striatal neuron ablation diminished the auditory fear conditioning only when the US was weak is intriguing since the striatum is supposed to play a role in incorporating the positive or negative value of information into the determination of behavioral responses.

## Results

### Generation of striatum-specific Cre mouse lines

**Figure 1:**
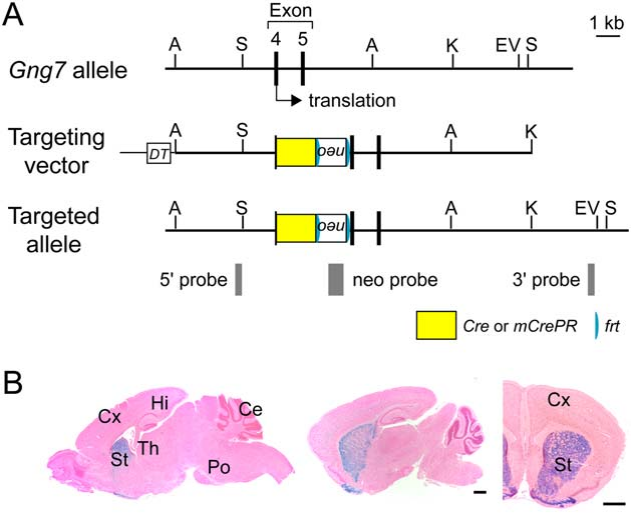
Generation of Gγ7-Cre and Gγ7-mCrePR mice. A, Schema of the exon 4 region containing the translational initiation site of the *Gng7* gene, targeting vector, and targeted allele. The targeting vector carries the *cre* or *mCrePR* gene and the *neo* gene flanked by two *frt* sequences. A, *Apa*I; EV, *Eco*RV; K, *Kpn*I; S, *Spe*I. B, *LacZ* expression following Cre recombination. X-gal-staining of sagittal and coronal sections from *Gng7^+/cre^*; *+/CAG-CAT-Z* mice at postnatal day 14. Sections were counterstained with nuclear fast red. Abbreviations: Ce, cerebellum; Cx, cortex; Hi, hippocampus; Po, pons; St, striatum; Th, thalamus. Scale bars, 1 mm.

The G-protein γ7 subunit mRNA is expressed predominantly in medium spiny neurons of the caudate-putamen (CP) and nucleus accumbens (NAc) and neurons of the olfactory tubercle [23]. To develop a striatal neuron-specific gene manipulation system, we produced Gγ7-Cre and Gγ7-mCrePR mouse lines by inserting the gene encoding Cre recombinase or Cre recombinase-progesterone receptor fusing protein (CrePR) into the translational initiation site of the G-protein γ7 subunit gene (*Gng7*) through homologous recombination in embryonic stem cells derived from the C57BL/6 strain [24] (Fig. 1A). We then crossed the Gγ7-Cre and Gγ7-mCrePR mice with the CAG-CAT-Z11 reporter mouse [25]. Brain slices prepared from Gγ7-Cre×CAG-CAT-Z11 mice were stained for β-galactosidase activity to monitor the Cre recombinase activity. Strong β-galactosidase staining was found predominantly in the CP, NAc and olfactory tubercle. Faint signals were detected in the layer 5 of the neocortex and subiculum (Fig. 1B). On the other hand, no β-galactosidase staining was detectable in brain slices from Gγ7-mCrePR×CAG-CAT-Z11 mice upon induction of Cre recombinase activity by RU-486 administration.

### Inducible ablation of striatal neurons

**Figure 2:**
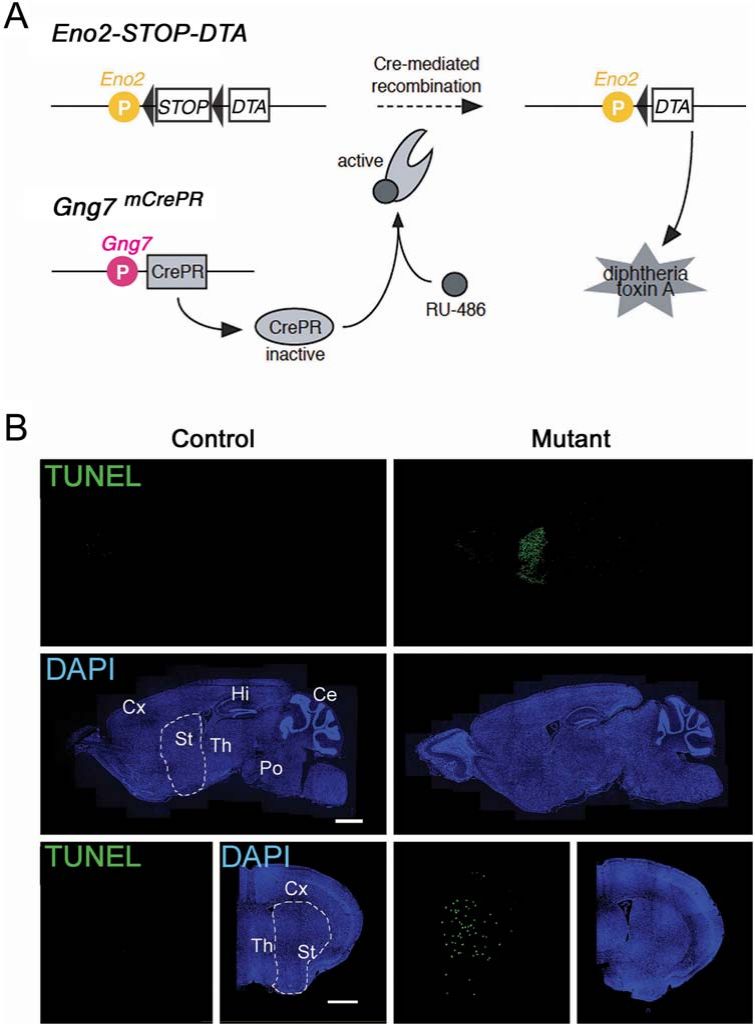
Inducible ablation of striatal neurons. A, Schema for striatal neuron ablation induced by RU-486 administration. B, TUNEL staining (green) counterstained with DAPI (blue) in brain sections of control (left) and mutant (right) mice 10 days after mock and RU-486 administration, respectively. Scale bars, 1 mm. Abbreviations: Ce, cerebellum; Cx, cortex; Hi, hippocampus; Po, pons; St, striatum; Th, thalamus.

We then crossed the Gγ7-mCrePR mouse with a knock-in mouse (Eno2-STOP-DTA) in which the Cre-inducible diphtheria toxin A gene (*DTA*) was introduced into the neuron-specific enolase gene (*Eno2*) locus [26]. In *Gng7^+/mCrePR^* mice, one allele retains the intact *Gng7* gene, and the other is inactivated by insertion of the *CrePR* gene. We injected 1 mg per g body weight of RU-486 into the peritoneum of Gγ7-mCrePR×Eno2-STOP-DTA mice at postnatal day 42 (P42) to induce the recombinase activity of CrePR [24], [25], [27] (Fig. 2A). Mock-injected mice served as controls. Ten days after RU-486 injection, TUNEL staining showed strong signals throughout the striatum, including the CP, NAc and olfactory tubercle (Fig. 2B). On the other hand, no TUNEL-signals were detectable in the striatum of the mock-injected mice. Both RU-486- and mock-treated mice showed faint TUNEL-signals in the olfactory bulb probably due to the turnover of adult-generated olfactory granule cells [28]. In addition, Gγ7-mCrePR mice exhibited no detectable TUNEL signals in the striatum upon RU-486 injection (data not shown). These results suggest that RU-486 treatment successfully induced recombination by CrePR, leading to cell ablation in the adult brain in the striatum-specific manner. Gγ7-CrePR-mediated recombination appeared to be critically dependent on target mice since β-galactosidase staining was hardly detectable in Gγ7-mCrePR×CAG-CAT-Z11 mice upon induction.

**Figure 3:**
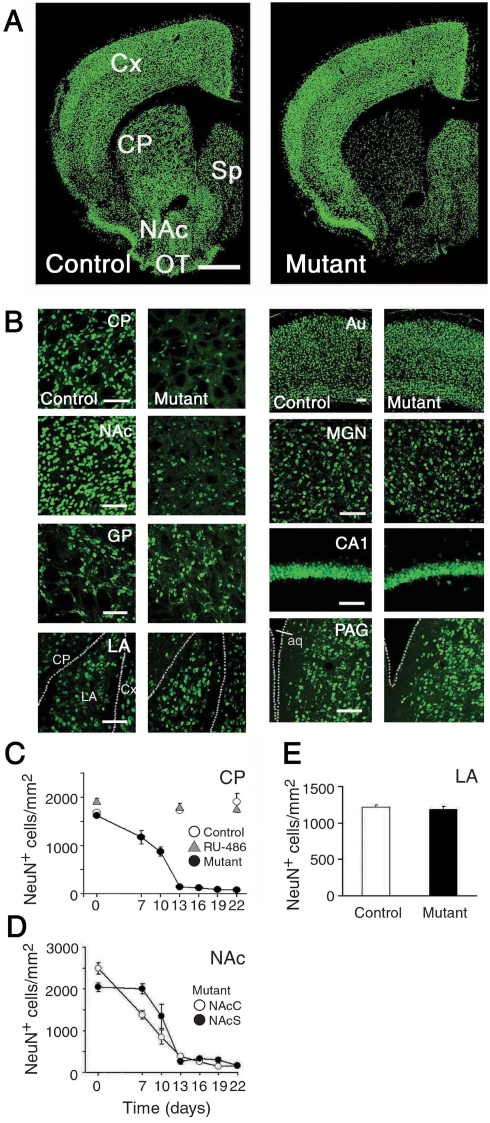
NeuN-immunohistochemstry. A, Immunohistochemical analysis for neuronal marker NeuN in control (left) and mutant (right) mice 13 days after mock and RU-486 administration, respectively. Scale bar, 1 mm. B, Higher magnification of NeuN-immunohistochemistry in various brain regions. Scale bars, 0.1 mm. C, NeuN immunoreactive (NeuN^+^)-cell density in the CP after drug administration. *n* = 8–9 each. D, Densities of NeuN-positive cells in the NAc core (NAcC, open circles) and the NAc shell (NAcS, filled circles) after RU-486 treatment of *Gng7^+/mCrePR^*; *+/Eno2-STOP-DTA* mice (*n* = 8–9 each). E, Densities of NeuN-positive cells in the lateral amygdala (LA) of control and mutant mice 22 days after mock and RU-486 treatment, respectively (*n* = 15 each, *F*_1,28_ = 0.23, *P* = 0.64, one-way ANOVA). Abbreviations: Au, auditory cortex; CA1, hippocampal CA1 region; CP, caudate putamen; Cx, cortex; GP, globus pallidus; MGN, medial geniculate nucleus of thalamus; NAc, nucleus accumbens; OT, olfactory tubercle; PAG, periaqueductal gray; Sp, septum.

Thirteen days after RU-486 treatment, TUNEL signals in the striatum became undetectable in Gγ7-mCrePR×Eno2-STOP-DTA mice. We then quantitatively examined the ablation of striatal neurons by immunohistochemical staining for NeuN, a marker protein for neurons. The density of NeuN-positive neurons in the CP drastically decreased by 13 days after RU-486 injection (*F*_6,54_ = 99.5, *P*<0.001, one-way ANOVA) and remained at a very low level thereafter (Fig. 3A–C). The number of NeuN-positive cells in the NAc core and shell also decreased with a similar time course (Fig. 3B,D). However, NeuN immunostaining signals in other brain regions including the amygdala were comparable between mock- and RU-486-treated mice (Fig. 3B,E).

**Figure 4:**
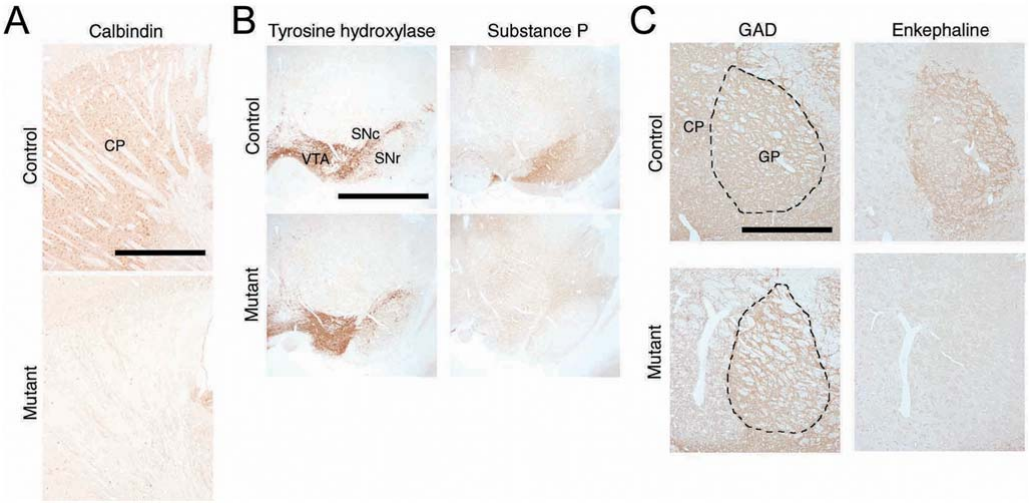
Ablation of medium-spiny projection neurons in the striatum of mutant mice. A, Immunoreactivity for calbindin in the dorsal striatum of control (upper) and mutant (lower) mice. B, Immunoreactivity for tyrosine hydroxylase and substance P in substantia nigra of control and mutant mice. C, Immunoreactivity for GAD and enkephalin in GP of control and mutant mice. Abbreviations: CP, caudate putamen; GP, globus pallidus; SNc, substantia nigra pars compacta; SNr, substantia nigra pars reticulata; VTA, ventral tegmental area. Scale bars, 1 mm.

Medium-spiny projection neurons, the main output neurons, account for up to 90% of neurons in the striatum [29], [30]. There were no detectable immunoreactivities for calbindin, a marker for medium-sized spiny neurons [31], in the mutant striatum (Fig. 4A). Medium-spiny projection neurons in the striatum can be largely subdivided into two groups: some that project to directly to the substantia nigra pars reticulata (SNr) (the direct pathway) express substance P; others that project to the same nucleus via the globus pallidus (GP) (the indirect pathway) express enkephalin [29]. These two neuropeptides are anterogradely transported to the axon terminals in the afferent regions [32]. There were no detectable immunoreactivities for substance P and enkephalin in SNr and GP, respectively, of RU-486-treated mice (Fig. 4B,C), suggesting that any striatal output scarcely remains in the basal ganglia of the mutant mice. Along with the NeuN-immunohistochemistry, our results suggest that induction of CrePR-mediated DTA expression by RU-486 injection successfully ablated almost completely the medium spiny neurons that comprise approximately 90% of the NeuN-positive striatal neurons within 13 days. In subsequent analyses, we used Gγ7-mCrePR×Eno2-STOP-DTA mice from 13 to 22 days after RU-486 administration as striatal neuron-ablated mutant mice and corresponding mock-injected littermates served as controls.

### Motor activity

**Figure 5:**
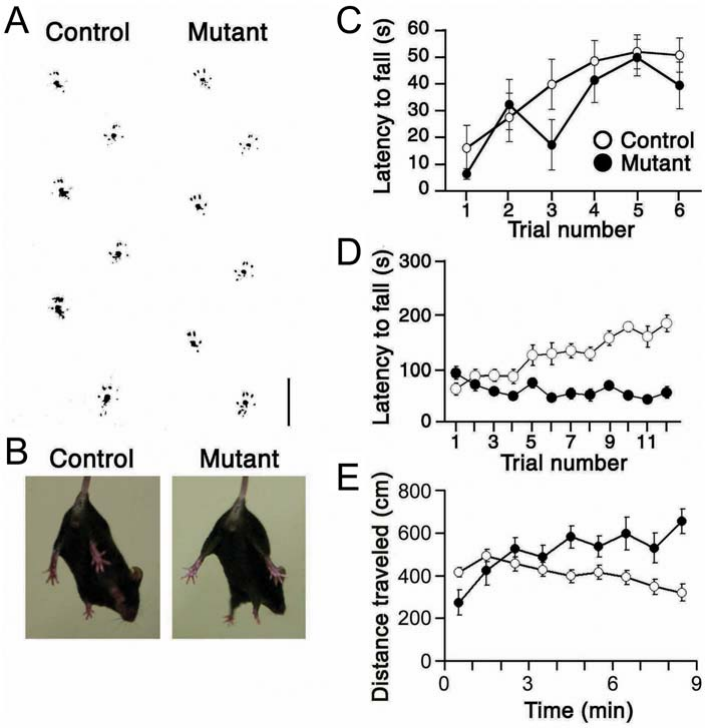
Performance of mutant mice in motor tests. A, Foot print of control (left) and mutant (right) mice. Scale bar, 2 cm. B, Tail suspension test of control (left) and mutant (right) mice. C, Performance of control (open circles, *n* = 9) and mutant (filled circles, *n* = 8) mice in the stationary thin rod test. D, Performance of control (open circles) and mutant (filled circles) mice in the accelerating rotarod (*n* = 8 each). E, Locomotor activity of control (open circles, *n* = 10) and mutant (filled circles, *n* = 7) mice in the openfield test.

The striatum is intimately involved in motor control. The striatal neuron-ablated mutant mice showed no ataxic gait or tremor and could walk along a straight line as control did (control, *n* = 4; mutant, *n* = 4) (Fig. 5A). There was no significant difference in the performance in the stationary thin rod test [33] between mutant and control mice (*F*_1,15_ = 1.38, *P* = 0.26, repeated measures ANOVA) (Fig. 5C). Thus, the ablation of striatal neurons appeared to exert little effect on motor coordination under standard conditions at least for a week after loss of ∼90% striatal neurons. In the accelerating rotarod test [34], both mutant and control mice performed equally well in the first training session (*F*_1,14_ = 3.57, *P* = 0.08, one-way ANOVA) (Fig. 5D). Despite that approximately 90% of striatal neurons were ablated, the motor performance of the mutant mice appeared to be comparable to that of control mice in stationary thin rod and rotating rod tests. In subsequent sessions of the accelerating rotarod test, however, there was a significant difference in the retention time between two groups (*F*_1,14_ = 37.2, *P*<0.001, repeated measures ANOVA). Control mice showed a steady and rapid improvement in their performance over the training. In contrast, mutant mice failed to exhibit any improvements over trials, suggesting that the striatal neurons are indispensable for motor learning. Our results are consistent with the observation that striatum-specific NMDA receptor mutant mice showed impaired motor learning in an accelerating rotarod test [35]. In the open field test, the locomotor activity of mutant mice tended to be higher than that of control mice (*F*_1,15_ = 4.6, *P* = 0.05) (Fig. 5E).

The degeneration of striatal neurons is associated with Huntington's disease [36], [37] and dystonia [38], [39]. Mutant mice, however, showed no abnormal clasping behavior induced by a tail suspension in a dystonic fashion (*n* = 6) (Fig. 5B); the clasping behavior was observed in the mutant mice 6 weeks after RU-486 injection. In addition, there were no easily recognizable movement disorders in mutant mice at least for a week after the drug-induced ablation of striatal neurons had been completed.

### Impairment of auditory fear conditioning with a low-intensity footshock

**Figure 6:**
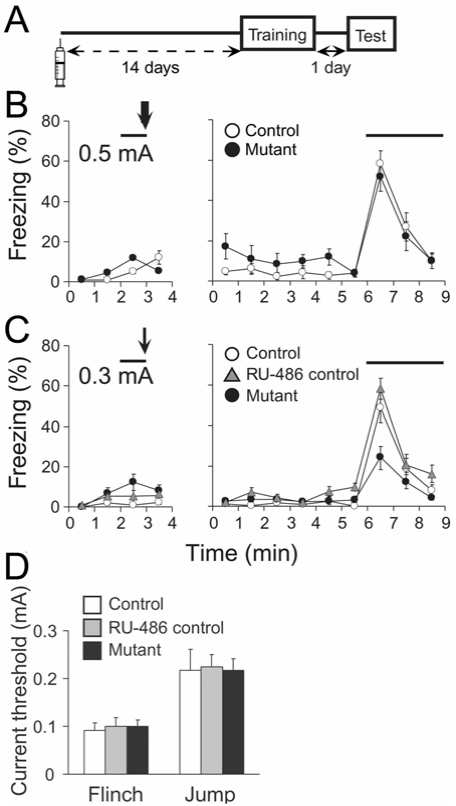
Impaired freezing responses of mutant mice after auditory fear conditioning with a low-intensity footshock. A, Experimental design. Mice were injected with RU-486 or vehicle. Fourteen days after treatment, the animals were subjected to auditory fear conditioning. B, Freezing responses of control (open circles, *n* = 9) and mutant (filled circles, *n* = 8) mice on the conditioning (left) and test (right) days. Auditory fear conditioning was carried out with the standard intensity of footshock (0.5 mA, an arrow). Solid lines represent tone. C, Freezing responses of control (open circles, *n* = 8) and mutant (filled circles, *n* = 11) mice and RU-486-treated Gγ7-mCrePR mice (RU-486 control) (shaded triangles, *n* = 7) on the conditioning (left) and test (right) days. Auditory fear conditioning was carried out with a low intensity of footshock (0.3 mA, an arrow). Solid lines represent tone. D, Current thresholds of control (open bar), RU-486-control (shaded bar) and mutant (filled bar) mice for flinch and jump reactions (*n* = 6 each).

Mutant mice were subjected to auditory fear conditioning to examine the possible involvement of striatal neurons in the formation of the emotional memory. Fourteen days after RU-486 treatment, mutant mice were trained for auditory fear conditioning (Fig. 6A). Mice were given a single pairing of tone (CS) and footshock (US; 0.5 mA) on the conditioning day (Fig. 6B). Twenty-four hours after the conditioning, the mice were placed in a novel chamber. Six min after placement, the tone was delivered for 3 min. Mice exhibited a range of conditioned fear responses including freezing. Levels of freezing during the pre-tone period were comparable between mutant and control mice (*F*_1,15_ = 2.28, *P* = 0.15). Freezing responses to the tone were also similar between mutant and control mice (control, 31.6±5.1%; mutant, 28.0±5.1%; *F*_1,15_ = 0.27, *P* = 0.61) (Fig. 6B). Thus, mutant mice successfully acquired fear memory under the standard condition despite of almost complete ablation of striatal medium spiny neurons.

We further investigated the ability of mutant mice to acquire fear memory under a less intensive condition. Mice were trained with a single paring of the tone and a low-intensity footshock at 0.3 mA, and tested for the freezing response 24 h after training. Negligible levels of freezing were observed during the pre-tone period in control and mutant mice as well as RU-486-treated Gγ7-mCrePR mice (RU-486 control). However, there were significant differences in the freezing responses across the CS presentation among 3 groups of mice (control, 29.7±4.9%; RU-486 control, 31.5±4.9%; mutant, 13.6±2.7%; *F*_2,23_ = 6.57, *P* = 0.006) (Fig. 6C). The freezing levels of mutant mice were much lower than those of control mice (*P*<0.05, mutant vs. control; *P*<0.01, mutant vs. RU-486 control; Post-hoc analysis). Comparable levels of freezing between control and RU-486-control mice indicated that treatment of RU-486 itself exerted little effect on the fear conditioning. There were no significant differences among control, RU-486 control, and mutant mice in pain thresholds for flinch and jump reactions (flinch, *F*_2,16_ = 0.094, *P* = 0.91, one-way ANOVA; jump, *F*_2,16_ = 0.021, *P* = 0.98) (Fig. 6D). The post-shock activity bursts [40] of mutant and control mice were also similar (at 0.3 mA, *F*_2,31_ = 3.30, *P* = 0.98; at 0.5 mA, *F*_1,13_ = 4.67, *P* = 0.23). These results suggest that striatal neurons are indispensable for efficient auditory fear conditioning with the low-intensity US.

### Impairment of long-term fear memory

**Figure 7:**
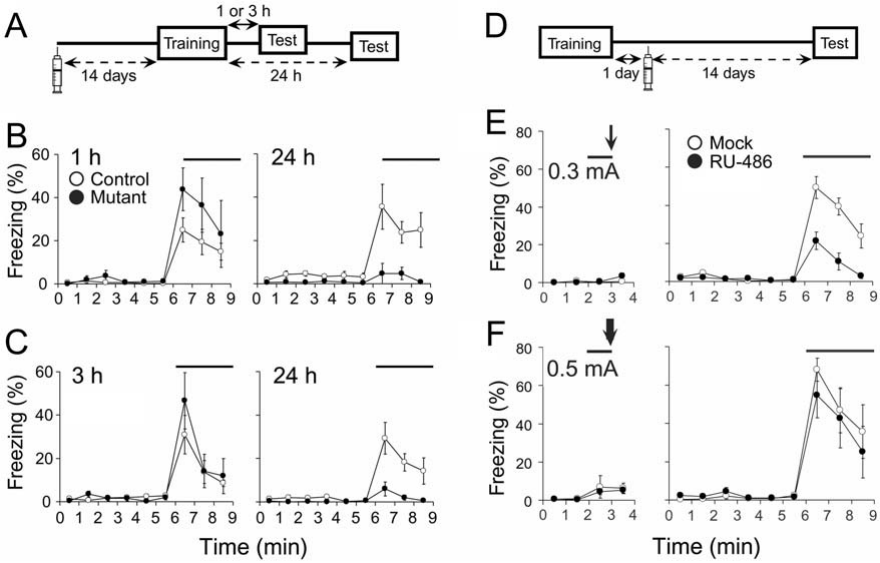
Impairment of long-term fear memory. A, Experimental design. Mice were injected with RU-486 or vehicle. Fourteen days after treatment, the animals were subjected to auditory fear conditioning with a weak footshock at 0.3 mA. Freezing responses to tone were measured 1 or 3 h and 24 h after conditioning. B, Freezing responses of control (open circles, *n* = 8) and mutant (filled circles, *n* = 5) mice 1 h (left) and 24 h (right) after conditioning. C, Freezing responses of control (open circles, *n* = 6) and mutant (filled circles, *n* = 4) 3 h (left) and 24 h (right) after conditioning. D, Experimental design. Mice were subjected to auditory fear conditioning with a footshock at 0.3 mA or 0.5 mA. One day after conditioning, the conditioned mice were injected with RU-486 or vehicle. Their freezing responses were measured 14 days after drug treatment. E, Mice were subjected to auditory fear conditioning with a weak footshock at 0.3 mA. Freezing responses of mock-injected (open circles, *n* = 7) and RU-486-injected (filled circles, *n* = 8) mice on the conditioning (left) and test (right) days. F, Mice were subjected to auditory fear conditioning with the standard footshock at 0.5 mA. Freezing responses of mock-injected (open circles, *n* = 6) and RU-486-injected (filled circles, *n* = 7) mice on the conditioning (left) and test (right) days.

To further examine the role of striatal neurons in fear conditioning, we trained mice under the weak condition (a single paring of tone and footshock at 0.3 mA), tested for short-term memory (STM) 1 or 3 h after training and then retested for long-term memory (LTM) 24 h after training [41] (Fig. 7A). The freezing responses of mutant mice were comparable to those of control mice 1 h after conditioning (control, 19.7±3.0%; mutant, 34.5±7.2%; *F*_1,11_ = 3.20, *P* = 0.10, repeated measures ANOVA) (Fig. 7B left panel) as well as 3 h after conditioning (control, 20.6±3.6%; mutant, 24.2±7.1%; *F*_1,8_ = 0.51, *P* = 0.50) (Fig. 7C left panel). Twenty-four hours after training, however, mutant mice showed significantly smaller freezing responses than control mice (Fig. 7B right panel, control, 28.0±4.6%; mutant, 3.4±1.8%; *F*_1,11_ = 8.06, *P* = 0.02: Fig. 7C right panel, control, 17.7±4.0%; mutant, 2.9±1.2%; *F*_1,8_ = 46.7, *P*<0.001). These results suggest that the striatal neurons are involved selectively in the acquisition of LTM under the weak conditioning, but not in that of STM. The intact STM formation in mutant mice is consistent with no detectable alterations in the sensitivity to the electric footshock as above.

### Impairment of fear memory retention

We further examined whether the ablation of striatal neurons affects the retention of previously acquired fear memory (Fig. 7D). Mice were first trained with a single paring of tone and footshock at 0.3 mA and placed back in the home cage. Twenty-four hours after conditioning when LTM was formed, the animals were treated with RU-486 for induction of striatal neuron ablation. When tested 14 days after the drug treatment, RU-486-injected mice showed significantly smaller freezing responses during tone presentation than mock-injected mice (mock-injected mice, 37.6±3.9%; RU-486-injected mice, 11.5±2.6%; *F*_1,13_ = 41.9, *P*<0.001) (Fig. 7E). On the other hand, the ability of RU-486-injected mice to retain the acquired fear memory under the standard condition (0.5 mA) was comparable to that of mock-injected mice (mock-injected mice, 50.1±6.8%; RU-486-injected mice, 40.8±8.0%; *F*_1,11_ = 0.32, *P* = 0.58) (Fig. 7F), consistent with the observation that pre-conditioning ablation of striatal neurons hardly affected the auditory fear conditioning (Fig. 6B). These results suggest that the striatal neurons are required for the retention of fear memory previously acquired by the conditioning with the low-intensity US.

## Discussion

Here, we show that striatal neurons can be selectively ablated upon induction in mice carrying *Gng7*-promoter-driven *CrePR* and Cre-dependent *DTA* genes. Despite that approximately 90% of striatal neurons were ablated, the motor performance of the mutant mice appeared to be comparable to that of control mice in stationary thin rod and rotating rod tests. However, the improvement of the mutant mice in the performance over trials was impaired in the accelerating rotarod test, suggesting the requirement of striatal neurons for motor learning. In addition, the mutant mice showed no abnormal behavior in the tail suspension test and there were no easily recognizable movement disorders in the mutant mice at least for a week after the drug-induced ablation of striatal neurons had been completed. Interestingly, however, the clasping behavior was observed 6 weeks after RU-486 injection. The motor phenotypes of mutant mice appeared later might be caused by secondary changes of the brain. It is known that dystonic symptoms occur a long time after brain injury, suggesting secondary changes [42], [43].

One to several pairings of tones with footshocks at 0.5–2 mA are generally used for fear conditioning in rodents [18]–[22]. The striatal neuron ablation hardly affected the auditory fear conditioning with a single pairing of tone with the footshock at 0.5 mA. Our results are consistent with previous ones that electrolytic or excitotoxic lesion of the striatum exerted little effect on the auditory fear conditioning [19]–[21]. On the other hand, a slight impairment of fear conditioning with 5 tone-footshock (0.5–1 mA) pairings was reported for dorsal striatum- or NAc shell-lesioned rats [44], [45]. It will be difficult to ascertain whether the discrepant behavioral effects of classical lesion studies were caused by ablation of striatal neurons or other impairments. Our genetic ablation system specific for striatal neurons provides evidence supporting the view that the amygdala but not the striatum is essential for the auditory fear conditioning under the standard condition.

In the present investigation, we found that when the tone was paired with the low-intensity footshock at 0.3 mA, the freezing responses 24 h after conditioning were significantly reduced in the striatal neuron-ablated mice. The impairment of tone-dependent fear conditioning with the low-intensity US itself does not reveal a specific role of these striatal neurons in either the learning or the performance of conditioned fear. However, the observation that the freezing responses of the mutant mice measured 1 h or 3 h after conditioning with the low-intensity US were comparable with those of control mice excluded the possibility that the striatal neuron ablation simply disrupted the animal's ability to make the freezing responses. Furthermore, the mutant mice showed the ability to acquire, retain and express the cued fear memory at least for 3 h after conditioning with the low-intensity US. It is to be noted with this respect that the induction of cell ablation was selective for striatal neurons, leaving the amygdala intact, which plays an essential role in the acquisition, storage and expression of fear memory [2], [18], [20]. Thus, the striatal neuron ablation appeared to impair the formation and/or retention of long-term fear memory rather than performance or acquisition and expression of fear memory. Consistently with this possibility, the ablation of striatal neurons after long-term fear memory formation, that is, 24 h after conditioning with the low-intensity US, diminished the retention of the LTM.

These results obtained by the use of an inducible striatal neuron-ablation system suggest the presence of at least two forms of the auditory fear memories distinguished by the US intensity and by the requirement of striatal neurons. Under the standard condition, auditory fear memory formation is hardly affected by the striatal neuron ablation, in agreement with previous studies showing that the amygdala but not the striatum plays a central role in the auditory fear conditioning [2], [18]–[21]. When auditory fear conditioning was carried out with the low-intensity US, the formation of LTM but not STM became sensitive to striatal neuron ablation. Thus, our results reveal a novel form of the auditory fear memory depending on striatal neurons at the low-intensity US. When the US becomes weaker, it will be less threatening and more difficult to judge whether it is dangerous enough to be memorized for animals. Our finding that striatal neuron ablation diminished the auditory fear conditioning only when a footshock was weak is of interest in view that the striatum is supposed to play a role in incorporating the positive or negative value of information into the determination of behavioral responses [46]–[48]. It is possible, though not proven, that striatal neurons may be activated by the weak US and directly or indirectly involved in the consolidation or retrieval of the long-term fear memory. While the contextual fear conditioning requires the hippocampus and amygdala, our results suggest further integration of brain systems for the emotional memory by showing the involvement of the striatum in the auditory fear conditioning at the weak US. Fear is an adaptive component of response to potentially threatening stimuli, but too much or inappropriate fear accounts for many common psychiatric problems, such as anxiety disorders [3]–[5]. Advances in basic and clinical neuroscience studies of fear are important for the development of strategies to treat and cure anxiety disorders [49], [50]. Thus, the finding of a novel form of the auditory fear memory might have therapeutic implications.

## Materials and Methods

### Generation of striatum-specific Cre mice

A full-length cDNA (210 bp) encoding the mouse G-protein γ7 subunit was amplified with primers 5′-GATGTCAGGTACTAACAACGTCGCCC-3′ and 5′-CTAGAGAATTATGCAAGGCTTTTTGTCTTT-3′ from a brain cDNA library from ICR mice. Using the cDNA fragment as a probe, we isolated a BAC clone containing the exon 4 and 5 of the *Gng7* by screening a genomic DNA library of C57BL/6 mouse (Genome systems, St. Louis, MO). The 8.9 kb-*Spe*I-*Kpn*I fragment from the BAC clone was inserted into the *Spe*I-*Kpn*I sites of pBluescript II SK(+) (Stratagene, La Jolla, CA) to yield pGng7MET. The 523 bp *Eco*RI-*Age*I fragment generated by 2-step-PCR using pGng7MET and pNCre [25] as templates and the 989 bp *Age*I-*Eco*RI fragment from pNCre were cloned into the *Eco*RI site of pBluescript II SK(+) to yield pGng7Cre. The 1.4 kb *Eco*RI-*Sac*I fragment from pGK1NeopA [27] was blunted and inserted into the *Eco*RV site of pGng7Cre to yield pCreNeo. Synthetic oligonucleotides (5′-AGCTTTCAGGTACTAACAACGTCGCCCAGGCCCGGAAGCTGGTGGAGCAGC-3′ and 5′-GCAGCTGCTCCACCAGCTTCCGGGCCTGGGCGACGTTCTTAGTACCTGAA-3′) and the 7.5 kb *Fsp*I-*Kpn*I fragment from pGngMET were ligated with the *Hind*III (blunted)- and *Kpn*I-digested pBluescript II SK(+) to yield pFSKP. The 7.5 kb *Eco*RV-*Kpn*I fragment from pFSKP was ligated with the *Hind*III (blunted)- and *Kpn*I-digested pCreNeo to yield pBTV. *Kpn*I-digested pMC1DTApA was blunted and ligated, and the 4.3 kb *Not*I-*Hind*III fragment from the resulting plasmid was ligated with synthetic oligonucleotides, 5′-GGCCGCGGTACCCGGGTCGACTTA-3′ and 5′-AGCTTAAGTCGACCCGGGTACCGC-3′, to yield pMC1DTApA2. The 11.1 kb *Not*I-*Kpn*I fragment of pBTV was inserted into the *Not*I-*Kpn*I sites of pMC1DTpA2 to yield targeting vector pGng7CreTV. The Cre coding sequence of the *CrePR* gene [25], [27] was replaced by that of mammalian Cre with the optimal codon usage in mammals by 2-step PCR using pNCrePR [25] and pCXN-Cre [51]. The 1.9-kb fragment encoding mammalian CrePR (mCrePR) was cloned into the *Xba*I site of pEF-BOS [52] to yield pmNCrePR. The *cre* gene in pGngCreTV was replaced by the *mCrePR* gene to yield targeting vector pGngmCrePRTV.

The targeting vectors were linearized by *Kpn*I and electroporated into ES cells derived from the C57BL/6 strain [24], [27]. Recombinant clones were identified by Southern blot analysis of genomic DNA using 0.25 kb *Age*I-*Spe*I fragment from the BAC clone, 0.6 kb-*Pst*I-*Pst*I fragment from pPGK1-NeopA and 0.3 kb *Nde*I-*Sac*I fragment from the BAC clone as 5′ outer, neo, and 3′ outer probes, respectively. Chimeric mice production was carried out essentially as described [24], [27]. The *Gng7^Cre^* allele was identified by PCR using primers CreP1 and CreP2 [25]. The *Gng7^mCrePR^* allele was identified by PCR using primers 5′-TATAGGTACCCAGAAGTGAATTCGGTTCGC-3′, 5′-GGCGACGTTGTTAGTACCTGAC-3′ and 5′-GTGCAGCATGTTCAGCTGGC-3′.

Eno2-STOP-DTA mouse [26] was backcrossed 7 times to the C57BL/6 strain. The *Eno2-STOP-DTA* allele was identified by PCR using primers 5′-AATTCTTAATTAAGGCGCGCGGG-3′, 5′-GTCAGAATTGAGGAAGAGCTGGGG-3′ and 5′-CACTGAGGATTCTTCTGTGG-3′. Breeding and maintenance of mice were carried out under institutional guidelines. Mice were fed *ad libitum* with standard laboratory chow and water in standard animal cages under a 12 h light/dark cycle. All animal procedures were approved by the Animal Care and the Use Committee of Graduate School of Medicine, the University of Tokyo (Approval #1721T062).

### Induction of CrePR recombinase activity

RU-486 (Sigma, St. Louis, MO) was suspended at a concentration of 50 mg/ml in water containing 0.25% carboxymethyl cellulose (Sigma) and 0.5% Tween 80 (Sigma). We injected 1 mg per g body weight of RU-486 into the peritoneum of mice at P42.

### Histochemistry

Under deep pentobarbital anesthesia (100 mg/kg), animals were perfused transcardially with 4% paraformaldehyde in 0.1 M phosphate buffered salts (PBS). β-Galactosidase staining was conducted as described previously [25]. Immunohistochemistry was performed as described previously [27] using antibodies against neuronal nuclei (NeuN), enkephalin, glutamic acid decarboxylase (GAD), substance P (Chemicon International, Temecula, CA), tyrosine hydroxyrase (Santacruz Biotechnology, Santa Cruz, CA), and calbindin. The numbers of NeuN-positive cells per 8.7×10^−2^ mm^2^ were counted at the dorsolateral part of CP, dorsomedial part of NAc core, the medial part of the NAc shell (AP = 1.2 mm from bregma), and the LA (AP = −1.7 mm) in the coronal brain sections. Only unequivocally stained cells were counted using the ImageJ software by two observers blind to the origin of the sections.

Terminal deoxynucleotidyl transferase-mediated dUTP nick-end labeling (TUNEL) histochemistry was performed using ApopTag Fluorescein Direct In Situ Apoptosis Detection Kit (Chemicon International) according to the instructions of the manufacturer. In brief, sections were incubated in PBS containing 20 µg/ml proteinase K (Ambion, Austin, TX) at room temperature for 15 min, washed and stained using FITC-labeled dUTP and terminal deoxynucleotide transferase (TdT) (Chemicon International) at 37°C for 60 min. After TUNEL reaction was terminated, slides were mounted using Vectashield H-1500 mounting solution that contains DAPI (Vector, Burlingame, CA). Confocal images were obtained using confocal microscopes (TCS-SP5, Leica, Wetzlar, Germany).

### Fear conditioning

A computer-controlled fear conditioning system (CL-M2; O'Hara, Tokyo, Japan) was used in the fear conditioning experiments. A clear conditioning chamber (10×10×10 cm) with polyvinyl chloride boards and a stainless steel rod floor that was composed of 14 stainless steel rods (2 mm in diameter spaced 7 mm apart) was surrounded by a sound-attenuating white chest (74 lux). Masking noise of 52 dB was provided by a ventilation fan. Mice were housed individually for 1 week before behavioral testing and were handled for 30 s everyday. On the conditioning day, mice were placed in the conditioning chamber for 2 min and then presented with a loud tone (65 dB, 10 kHz) for 1 min through a speaker on the ceiling of the conditioning chest. At the end of the tone presentation, the mice were given a scrambled electrical footshock (0.3 mA or 0.5 mA for 1 s). One minute after footshock, the mice were returned to their home cages. The conditioning chamber was cleaned with 70% ethanol between sessions. On the test day, mice were placed in a novel translucent acryl chamber with paper chips surrounded by a sound-attenuating black chest for 6 min and subsequently for 3 min in the presence of the tone. The test chamber was cleaned with benzalkonium (Ecolab, St. Paul, MN) between tests. All behaviors were monitored by a CCD camera (WAT-902B; Watec, Yamagata, Japan) attached to the ceiling of the chest. Eight bit grayscale images (90×90 pixels) were captured at a rate of two frames per second and freezing behavior was automatically analyzed as an index of fear using IMAGE FZC software (O'Hara). Freezing behavior was defined as the absence of any visible movement of the body and vibrissae except for movement necessitated by respiration. Freezing time was summated and the percentage of freezing was calculated per minute. To examine pain sensitivity, we measured current thresholds for reactions of mice to nociceptive shock, namely, flinch and jump [53]. Mice were given footshocks of increasing strength ranging from 0.05 to 0.5 mA in a stepwise manner by 0.05 mA with an interval of 30 s.

### Motor behaviors

The stationary horizontal thin rod test was done as described [33]. The rod was 15 mm in diameter and 50 cm long and placed 40 cm high to discourage jumping. A mouse was placed on the midpoint of the rod, and the time it remained on the rod was measured; animals staying for 60 sec were taken from the rod and recorded as 60 s. Six consecutive trials were performed with an intertrial interval of 1 h.

The accelerating rotarod test was performed with an apparatus consisted of a 3.2 cm-diameter rod (RRAC-3002; O'Hara & Co., Tokyo, Japan) essentially as described [34]. During the training period, mice were placed on the rotating rod starting at 5 rpm and gradually accelerated to 50 rpm at a rate 0.15 rpm/s. The latency to fall (retention time) was measured with cutoff time of 5 min. Mice were trained for 3 consecutive days, receiving 4 trials per day with an intertrial interval of 1 h.

In the tail suspension test [54], mice were observed for 15 s. Abnormal movement was defined as any dystonic movement of the hindlimbs, forelimbs, or trunk with full clasping where limbs were pulled into the central body axis.

In the open field test, locomotor activity was measured for 9 min in a square chamber (50×50×40 cm) with a CCD camera on the ceiling (OF4, O'Hara). Images were captured at a rate of one frame per second and walking distance was automatically measured by IMAGE OF4 software (O'Hara).

### Statistics

Data are expressed as mean±SEM. The statistics significance was evaluated using one-way or repeated measures ANOVA. When the interaction was significant, Fisher's PLSD post hoc test was employed. The criterion for statistical significance was *P*<0.05.
